# Hyponatremia due to Severe Primary Hypothyroidism in an Infant

**DOI:** 10.3389/fped.2015.00096

**Published:** 2015-11-10

**Authors:** Nickolas T. Agathis, Ingrid M. Libman, Michael L. Moritz

**Affiliations:** ^1^Texas Children’s Hospital, Baylor College of Medicine, Houston, TX, USA; ^2^Children’s Hospital of Pittsburgh of UPMC, The University of Pittsburgh School of Medicine, Pittsburgh, PA, USA

**Keywords:** child development, hyponatremia, hypothyroidism, infant, newborn, thyroxine

## Abstract

Hyponatremia has been reported in the elderly with hypothyroidism and myxedema, but this has not been a universal finding in clinical studies and there have been only a few reports in children. We report a case of an infant who developed hyponatremia due to severe primary hypothyroidism. A 4-month-old ex-preterm male, who had been euthyroid on the newborn screen, developed unexplained hospital-acquired hyponatremia (serum Na 124 mEq/L) while on full oral feeds. He was euvolemic, appeared well and was without myxedema. An evaluation of hyponatremia was negative with the exception of severe primary hypothyroidism (TSH 315.4 IU/mL, repeat 540 IU/mL). The hyponatremia resolved with thyroxine supplementation. This case demonstrates that severe hypothyroidism can result in hyponatremia in infants. It is critical to consider hypothyroidism in the evaluation of an infant with unexplained hyponatremia as untreated hypothyroidism can lead to profound developmental delays.

## Introduction

Hyponatremia is the most common electrolyte abnormality encountered in hospitalized patients. It is primarily the result of excess free water intake in conjunction with impaired free water excretion due to arginine vasopressin (AVP) excess. One potential cause of hyponatremia is hypothyroidism. This association has been primarily reported in the elderly with myxedema (1, 2), with only a few reports in children (3–6). The mechanisms linking the two entities are not entirely clear, but there is evidence to support both prerenal and renal mechanisms. There is controversy in the literature whether hypothyroidism produces hyponatremia, as this has not been a universal finding in all studies.

We report a 4-month-old ex-preterm male with a complicated past medical history and who was reported to have a normal newborn screen, who developed unexplained hyponatremia. This led to the diagnosis of severe hypothyroidism with resolution of the hyponatremia following thyroxin supplementation.

## Case Report

A 4-month-old male was born at 32-week gestation as a result of premature rupture of membranes with a birth weight of 1782 g and APGAR scores of 3 and 8 at 1 and 5 min postdelivery, respectively. His newborn screen was reported as normal. His past medical history was significant for an omphalocele repaired at 2 days of life. Between 14 and 15 weeks of life, he had a pyloric stenosis treated with a pyloromyotomy, gastroesophageal reflux treated with a Nissen fundoplication and gastrostomy tube, and a bilateral herniorrhaphy. Other complications included influenza-A pneumonia during the first month of life requiring 17 days of mechanical ventilation, chronic lung disease, and a grade I intraventricular hemorrhage noted at 6 weeks of age on head ultrasound, which had resolved at 4 months of age. An echocardiogram revealed a resolved patent ductus arteriosus with a clinically insignificant patent foramen ovale and a normal renal sonogram.

At 16 weeks of age, weight 4.57 kg, having been admitted to the hospital since birth, he was switched from total parenteral nutrition to enteral feeds consisting of Neocate and breast milk. At that time, his serum sodium was 136 mEq/L. Serum chemistries were done 4 days later, which revealed a serum sodium level of 124 mEq/L and plasma osmolality of 260 mOsm/kg confirmed on two repeated measurements (Table 1). There was no apparent explanation for the hyponatremia, since there was no apparent volume depletion, vomiting, diarrhea, or gastrostomy tube loss. He was stable from a respiratory standpoint and was not requiring oxygen. His only medications were famotidine, lansoprazole, and methadone for fentanyl withdrawal. He was not receiving diuretics or intravenous fluids. He was neurologically asymptomatic without lethargy, irritability, vomiting, or feeding intolerance. His total feeds in the previous 24 h were 110 mL/kg and his weight had increased by 400 g since the previous sodium level 4 days earlier.

**Table 1:** **Laboratory investigations**.

	Day -4	Day 0	Day 1	Day 2	Day 4	Day 7	Day 73
**SERUM (REFERENCE RANGES)**							
Na (mEq/L)	136	124	138	133	136	134	137
K (mEq/L)	5.2	4.9	5.3	5.4	5.4	6.3	6.3
Cl (mEq/L)	106	88	103	100	107	97	96
CO_2_ (mEq/L)	23.0	31.0	26.0	28.0	26.0	29.0	34
BUN (mEq/L)	25	15	12	13	19	21	16
Cr (mg/dL)	0.3	0.2	0.2	0.2	0.2	0.3	0.2
Glucose (mg/dL)	89	69	94		100	62	
Uric acid (mg/dL)		0.9			1.4		
Osmolality (mOsm/kg)		260					285
Cortisol (ug/dL) (3–23)		11.5					2.2
TSH (IU/mL) (1.7–9.1)		315.4		539.86		92.9	0.27
T3 (ng/dL) (1.03–2.29)		0.85		1.50		2.50	
T4 (ug/dL) (7–15)		2.3		5.8		12.3	
Free T4 (ng/dL) (0.8–1.8)				0.52		2.09	
**URINE**							
Specific gravity		1.006					
pH		8.0					
Osm (mOsm/kg)		389			349		172
Na (mEq/L)		23			63		21
K (mEq/L)		57.2			39.2		15.8
Cl (mEq/L)		32			69		<15
Cr (mg/dL)		15			14		6
Uric acid (mg/dL)		30.3			21.4		9.4
FENa (%)		0.2			0.7		0.5
FEUrate (%)		45			22		18

An evaluation of hyponatremia included serum and urine biochemistries including liver function tests, thyroid function tests, a cortisol level, serum and urine osmolality levels, and a uric acid level (Table 1). Spot urine electrolytes revealed a low urine sodium level of 23 mEq/L and a low fractional excretion of sodium (FENa) of 0.2%; this combination of findings was suggestive of a prerenal state. He had severe hypouricemia with a uric acid level of 0.9 mg/dL and an elevated fractional excretion of urate (FEUrate) of 45%. This combination of findings was suggestive of a Syndrome of Inappropriate Antidiuretic Hormone secretion (SIADH)-like state. Thyroid function tests revealed severe hypothyroidism (TSH 315.4 IU/mL) (normal range 1.7–9.1 IU/mL), which was confirmed on repeat measurement with a free T4 of 0.52 ng/dL (normal range 0.8–1.8 ng/dL) and a TSH of 540 IU/mL, with a normal cortisol. The hyponatremia was initially corrected with a 24-h infusion of 0.9% sodium chloride at a rate of 4 mL/kg/h and thyroid supplementation, to a sodium level of 138 mEq/L. The hypothyroidism was treated with 37.5 mcg daily of levothyroxine on Day 1 and 2, which was increased to 50 mcg daily on day 3. The intravenous fluids were discontinued on day 1 and the sodium level decreased to 133 mEq/L on day 2. The serum sodium then normalized without further intravenous fluids and without oral sodium supplementation. On day 4, repeat urine and serum chemistries revealed a serum sodium of 136 mEq/L, a spot urine sodium of 63 mEq/L with FENa of 0.7% and resolving hypouricemia with an FEUrate that decreased to 22% and eventually to 18% on day 73 (Table 1). Due to mild hyperkalemia an ACTH stimulation test was done which was normal.

Family history was remarkable for hypothyroidism in the maternal grandmother and a paternal aunt. To further evaluate the cause of hypothyroidism, a thyroid ultrasound and thyroid scan were done, which were both normal. The scan demonstrated homogeneous uptake at the expected location of the thyroid gland on both sides of the neck with no heterotopic uptake seen. Thyroid autoantibodies and genetic testing were not performed. His levothyroxine requirements decreased overtime and at 2-year follow-up, he was on 25 mcg daily. His development at 2 years of age is almost normal with the exception of an oral aversion requiring gastrostomy tube feedings and mild speech delay.

## Discussion

We report a case of an ex-preterm infant found to have hospital-acquired hyponatremia most likely due to hypothyroidism as other causes of hyponatremia were excluded. While hyponatremia from SIADH can be due to medications, methadone and lansoprazole are extremely infrequent causes of hyponatremia and the hyponatremia resolved promptly following treatment of the hypothyroidism despite continuing these medications. The exact etiology of the hypothyroidism has not yet been identified. Congenital hypothyroidism can not be excluded as the newborn screen for congenital hypothyroidism may have given a false negative result as the child was an ex-preterm infant of 32-week gestation and was also ill requiring an omphalocele repair at 2 days of age. Also the neonatal screening was not repeated 2 weeks after the first screening as recommended by consensus guidelines (7). The associated features of omphalocele, pyloric stenosis, and cardiac anomalies are suggestive of a genetic cause, which would merit further evaluation. Acquired hypothyroidism is unlikely; however, thyroid autoantibodies were not checked.

The association between hyponatremia and hypothyroidism has been recognized primarily in adults with severe myxedema (1, 2), but is an extremely unusual finding in an infant. There have been four previous reports in children (3–6), three of whom were infants with congenital hypothyroidism, of which one was water intoxicated (5), and another an 8-year-old child with hypothyroidism as a result of brain injury from status epilepticus (4).

There are two possible mechanisms described in the literature in which hypothyroidism could lead to hyponatremia; our patient had features of both. The prevailing view is that of a prerenal mechanism and compensatory dilutional hyponatremia (8). It has been shown that hypothyroidism initially causes a significant increase in peripheral vascular resistance, decrease in cardiac output, and concomitant decrease in glomerular filtration rate (8). There is also evidence to suggest impaired sodium reabsorption in the proximal and distal tubules as a result of decreased Na–K ATPase activity; this also would lead to volume depletion and aggravate the pre-renal mechanisms (9, 10). Supporting this hypothesis are studies, which have demonstrated a relationship between hypothyroidism and subclinical and clinical pre-renal acute kidney injury (11). Effective circulating volume depletion from hypothyroidism would lead to up-regulation of both the rennin–angiotensin–aldosterone system and AVP, leading to hyponatremia.

Another possible mechanism of hyponatremia due to hypothyroidism is the SIADH. Some studies have demonstrated inappropriately elevated AVP levels, possibly due to impaired osmoregulation or decreased metabolic clearance of AVP in hypothyroid patients (12).

The pathogenesis of hyponatremia in this patient appears to be most consistent with the theory of a pre-renal state and concomitant proximal tubular dysfunction. Our patient’s FENa was low at 0.2% with an elevated BUN-to-creatinine ratio of 80, reflective of appropriate renal compensation secondary to decreased effective circulating volume. The decreased urinary sodium excretion might also partially reflect decreased sodium intake as the patient was on a low sodium enteral diet. Patients with SIADH can have a low urine sodium concentration and FENa, if they are sodium restricted (13). Our patient also had significant hypouricemia and an elevated fractional excretion of urate of 45% (normal 13–26%) (14), which is consistent with proximal tubular dysfunction from natriuretic peptides (15). This phenomenon may also be encountered in SIADH and cerebral salt wasting (16). After levothyroxine was started, the hyponatremia, hypouricemia and elevated fractional excretion of urate resolved. This suggests that the hyponatremia was related to hypothyroidism.

This case report supports the assertion that severe hypothyroidism is associated with hyponatremia, yet other investigators have not been able to find an association. In a sample of 445 hypothyroid adults, the frequency of hyponatremia was no different than in euthyroid controls (17). Similarly, there was no difference in sodium concentrations or the incidence of hyponatremia in a homogenous group of 32 congenital hypothyroid infants compared to age matched controls (18). Warner et al. was able to demonstrate a statistically significant, but seemingly clinically insignificant, relationship between hypothyroidism and hyponatremia, with a fall in serum sodium concentration of 0.14 mEq/L for every 10 IU/L rise in TSH (19). Based on these findings, our patient’s TSH of 539 IU/L is associated with an average fall in serum sodium concentration of approximately 7 mEq/L from baseline. Therefore, this model was partially accurate in predicting that our patient could develop clinically significant hyponatremia.

This case report supports the association between hypothyroidism and hyponatremia and supports the physiologic hypothesis of extracellular volume depletion with proximal tubular dysfunction. While hyponatremia may be an uncommon consequence of severe hypothyroidism in infants, it is nevertheless an important cause. Hypothyroidism in infancy can lead to significant neurological damage and delays in intellectual development, which can be prevented with appropriate thyroxine treatment (20, 21). Therefore, if not for the development of hyponatremia in this patient, the diagnosis of hypothyroidism would likely have been missed or delayed leading to possible irreversible neurological damage and developmental delay in our patient. We therefore recommend that thyroid function tests be included in the evaluation of unexplained hyponatremia, particularly in the pediatric population. The test is of minimal cost and could rule out an easily treatable condition with significant morbidity.

## Ethics Statement

A formal consent is not required for case reports at our institution. Verbal consent was obtained prior to working on the case and was again obtained after completion of the report.

## Conflict of Interest Statement

The authors declare that the research was conducted in the absence of any commercial or financial relationships that could be construed as a potential conflict of interest.

## Abbreviations

AVP, arginine vasopressin; BUN, blood urea nitrogen; FENa, fractional excretion of sodium in the urine; FEUrate, fractional excretion of urate; TSH, thyroid stimulating hormone.
